# St. George's Hospital and King Edward's Fund

**Published:** 1912-06-01

**Authors:** A. William West

**Affiliations:** Treasurer and Chairman. St. George's Hospital, S.W.


### St. George's Hospital and King Edward's Fund.

LETTER FROM THE TREASURER OF ST. GEORGE'S HOSPITAL.

A COMMITTEE PROPOSED.

To the Editor of *The Times*.

SIR,—In my reply to the letter of the honorary secretaries of the King's Fund of May 14 I did not quote the final part of the letter of May 6, 1909, as it did not seem to me to have any further bearing upon the point that St. George's Hospital had received an intimation that the change in the accounts of the hospital could not be accepted without investigation; neither, taking the letter as a whole, can I see that there is any intimation to that effect; but I again assert that upon this letter the House Committee of St. George's Hospital was entitled to assume that, as the payment was "a specific payment for definite work done in respect of the treatment of patients," it was properly, and with the approval of the Fund, taken out of the general account.

The honorary secretaries appear to imply that St. George's Hospital did not carefully consider the apportionment between hospital and school of the maintenance of laboratories, but simply worked out the figures so as to produce a similar result. This is not the case, and only shows how carefully the matter was considered and how near the correct apportionment was.

The deputation that waited upon the King's Fund did not state "that the charges to the hospital were based on the amount the hospital would have had to pay for maintaining the laboratories necessary for its purposes if there were no school to share the cost of them and consequently to bear a portion of the cost." It was only pointed out that, if there were no school to bear this share of the cost of maintenance of laboratories, and the same amount of work was to be undertaken, the cost to the hospital would be far greater.

The honorary secretaries I regret in no way refer to the real difficulty, which is their assertion that St. George's Hospital spends two and a-half to two and three-quarter times above the average of other hospitals upon certain work. They can, if they are so pleased, produce the figures upon which they base this statement and which St. George's Hospital, after very carefully going into the whole matter, do not believe could be substantiated if all the facts are taken into consideration.

As I have previously stated, the whole of this difficulty has arisen upon the question of cost. St. George's Hospital maintains that it does not give anything towards medical education, either directly or indirectly, and that, if the whole of the circumstances are taken into account, it does not spend above the average of other hospitals upon certain branches of its work.

When two parties cannot agree upon such a matter, is it not fair to propose that some independent committee should go into the question and hold an inquiry into all the circumstances?

The King's Fund and St. George's Hospital are working with exactly the same object in view—to spend their money to the best advantage for the benefit of the sick poor, and I do trust that the Fund will be able to see its way to accepting this proposal, as they have not produced the figures upon which their whole argument rests.

Yours faithfully,

A. WILLIAM WEST, Treasurer and Chairman.

St. George's Hospital, S.W., May 22.

